# Dental implant survival in epidermolysis bullosa patients: A systematic review conducted according to PRISMA guidelines and the Cochrane handbook for systematic reviews of interventions

**DOI:** 10.1016/j.heliyon.2024.e24208

**Published:** 2024-01-07

**Authors:** Giuseppe Minervini, Rocco Franco, Maria Maddalena Marrapodi, Antonino Lo Giudice, Marco Cicciù, Vincenzo Ronsivalle

**Affiliations:** a Multidisciplinary Department of Medical-Surgical and Dental Specialties, University of Campania Luigi Vanvitelli, Naples, Italy; b Department of Biomedicine and Prevention, University of Rome “Tor Vergata”, Rome, Italy; c Department of Woman, Child and General and Specialist Surgery, University of Campania “Luigi Vanvitelli”, Naples, Italy; d Department of Biomedical and Surgical and Biomedical Sciences, Catania University, 95123, Catania, CT, Italy

**Keywords:** Epidermolysis, Dental implant, Skin disease

## Abstract

**Background:**

Epidermolysis bullosa (EB) is a genetic syndrome afflicting skin and mucous membranes. The manifestation depends on the form: in mild conditions, occasionally, vesicular-bullous lesions of the oral cavity may be present, which heal spontaneously without leaving scars. Patients following joint rupture have scars that limit food intake and restrict quality of life. This study aims to evaluate the possibility of carrying out an implant therapy and the success rate of this therapy.

**Methods:**

Until January 3, 2000, PubMed, Web of Science, and Lilacs were searched. Clinical studies were selected that considered implant therapy in patients with epidermolysis bullosa. Articles were therefore selected that addressed oral health and implant survival in patients with epidermolysis, with no differentiation between the various subtypes. A risk of bias assessment was performed through Cochrane.

**Results:**

Twenty-one studies were found after the investigation. Only five were chosen to create the current systematic study; 16 articles were skipped over. 10 papers were disregarded because they had been reviewed; 4 were ignored because they contained case studies; and two were omitted because they were not written in English. The results show that implant survival is at around 97%.

**Conclusions:**

Patients with epidermolysis bullosa can be treated with implant therapy without the risk of an increased implant failure rate. Indicate the main conclusions or interpretations.

## 1 Introduction

Epidermolysis bullosa (EB) is a skin disease that causes skin fragility with blistering following minimal mechanical trauma [1,2]. All this leads to the rupture of the dermo-epidermal junction, causing significant scars in many cases disabling and the development of aggressive squamous cell carcinomas. EB is a genetic syndrome [3,4]. The latest classifications of EB recognize four hereditary types due to the mutation of 20 distinct genes. EB is classified into four types: EB simplex (EBS), junctional EB (JEB), dystrophic EB (DEB), and Kindler EB (KEB). Other hereditary disorders are characterized by skin fragility, but the formation of blisters is not predominant. However, more superficial skin erosions are present. EB occurs in populations all over the globe and affects both sexes. Eight cases per 1,000,000 people and 19 cases per 1,000,000 live births are the estimated prevalence and incidence of the illness, respectively, in the United States. The rates in the United States are comparable to those in Western Europe and are presumably applicable globally.9 Researchers have discovered significant variations in stated prevalence across nations, with South Africa and Scotland reporting prevalence rates of two to 56 patients per 1,000,000 people, respectively. These discrepancies could be due to incomplete data entry into official EB databases, regional variations, different genetic susceptibilities, or a mix of these. Nearly a century later, in 1989, EB specialists gathered to discuss an agreement regarding the identification and categorization of inherited EB. Experts have suggested a classification scheme based on the degree of skin cleavage in the ultrastructure. The new method allowed EB to be divided into three main groups: junctional EB (JEB), EB simplex (EBS), and dystrophic EB (DEB).

The suggested classification system divided EB into subgroups based on the genetic mode of transmission and clinical phenotype.

In 2007, Vienna hosted the third and most recent consensus conference on the diagnosis and classification of EB. The inclusion of Kindler syndrome as a fourth major group of EB and the division of the condition into 32 subtypes was subsequent.

In addition to the inherited genetic forms, there is a variant called acquired epidermolysis bullosa [[5], [6], [7], [8]]. It is classified as an autoimmune bladder-forming disease caused by autoantibodies against type VI collagen in the dermo-epidermal junction. This disabling pathology has no cure; however, several unsubstantiated therapies are being evaluated. Clinical manifestations vary depending on the EB subtype. Depending on the subtype, hereditary EB is a dominant or recessive autosome genetic disorder. However, some ex-novo mutations, not uncommon, cause the onset of the pathology. Incomplete penetration is also quite common. EB is caused by the mutation of one of the structural proteins in keratinocytes or proteins in the dermo-epidermal junction [[9], [10], [11], [12], [13], [14], [15], [16]]. Therefore, based on the mutated protein, we have different clinical manifestations and subtypes of EB. Alterations of the keratin gene cause localized EBS, DEB, and other generalized EBS. The mutation's site determines the disease's severity and clinical manifestations. The oral presentations of EB vary depending on the severity of the disease; in mild forms, occasional vesicular-bullous lesions of the oral cavity may be present, which heal spontaneously without leaving scars. Therefore, the quality of life of the patient is not affected. Depending on the EB subtype, the oral manifestations of EB can affect both soft and hard tissues, and they vary in frequency and intensity. The most typical oral finding is blistering despite no pathognomonic intraoral soft tissue symptoms. Bullae burst, leaving behind painful ulcers, scarring, and tissue contraction. Milia, microstomia, ankyloglossia, serious periodontal disease, enamel hypoplasia, dental caries, and maxillary atrophy with mandibular prognathism are some additional symptoms. Oral squamous cell carcinoma, which rises with age, is more likely to occur in patients with DEB. Microstomia, which causes scarring in the commissures and the loss of vestibular space, is a continuous process of blister formation and healing that causes a narrowing of the mouth's opening. It hinders access to the mouth and gastrointestinal tract and adds to poor oral intake. Ankyloglossia, which restricts tongue movement and makes it difficult to swallow and consume, is caused by adhesions that develop between the tongue and the floor of the mouth. Patients with JEB or DEB were found to have a high risk of developing caries, according to Wright and coworkers [17]. The prevalence of caries in patients with EBS or DEB, however, was comparable to that of healthy control participants, according to other reports. The occurrence of dental plaque (as a result of poor oral hygiene caused by the discomfort of brushing and the fear of developing new lesions), pseudosyndactyly, microstomia, a diet high in soft, carbohydrate-rich foods, and decreased oral clearance are factors that favour caries formation in these patients. Although the mineral and chemical makeup of tooth enamel in DEB patients is the same as that of normal enamel, the developmentally compromised enamel found in JEB patients may also put them at risk of cavities. In severe cases, oral manifestations can be life-threatening due to blisters and consequent scarring that cause microtomy, lowering of the vestibule, and ankyloglossia [[18], [19], [20]]. Therefore, these patients struggle to take solid food and remove oral biofilm. Depending on the type of EB, teeth may also be affected, with enamel hypoplasia and dental abnormalities [21]. Clinical manifestations are related to the type of EB, therefore, based on altered protein. In DEB is impaired type IV collagen that is essential for the integrity of the skin and oral mucosa but does not affect the teeth; instead, in some types of JEB where laminin is involved, the teeth are severely involved with very severe hypoplasia of enamel [22]. The production of saliva is not altered. Therefore, an increase in the caries index is not due to the decrease in saliva but to problems with the structure of the enamel and alterations in diet [23,24]. Some dental implant (DI) systems have documented success rates of between 90 and 95% at ten years in medically sound patients. However, DI may fail because of a lack of osseointegration during the first stages of recovery or while the implant is functioning because it breaks or becomes infected, which results in the loss of implant support. Pain, infections, and occasionally neuropathy are possible early complications following implant placement. Rarely do patients experience severe early consequences like haemorrhage (for example, in the mouth floor) or descending necrotizing mediastinitis. Local or systemic illnesses or other limiting factors may impact the longer-term results of implant therapy; in fact, some local and systemic factors may be contraindicated for DI treatment. Numerous case studies and case series demonstrate the effectiveness of DI in patients with various mucosal conditions, including lichen planus, epidermolysis bullosa, and ectodermal dysplasia [25].

When a patient has severe oligodontia or hypodontia due to ectodermal dysplasia, DI is frequently the recommended course of therapy. It was expected to discover that only a small amount of bone was present, especially in the upper arch, which frequently necessitated extensive bone regenerative procedures. According to some case studies, the outcomes of DI and bone transplants in adult ectodermal dysplasia patients were comparable to those of unaffected patients [26,27].

While malocclusion and EB are not directly related, individuals with EB may face challenges related to oral health. Blisters and erosions in the mouth can contribute to difficulties in maintaining proper oral hygiene, leading to an increased risk of dental issues, including malocclusion. Additionally, scarring from chronic blistering in the oral cavity may impact the development and alignment of teeth.

In managing oral health in individuals with EB, a multidisciplinary approach involving dermatologists, dentists, and other healthcare professionals is crucial. Special attention is given to preventive measures, such as regular dental check-ups, to address and mitigate any potential issues related to malocclusion.

In summary, malocclusion and Epidermolysis Bullosa are distinct medical conditions, but individuals with EB may face unique challenges in maintaining oral health that can indirectly contribute to dental issues like malocclusion. It underscores the importance of comprehensive care and collaboration among different healthcare specialists in managing the overall well-being of individuals with complex medical conditions. Although results reported in children and teenagers, primarily when implants were placed in the maxilla or the symphyseal region of the anterior mandible, have been less encouraging, most series show an excellent implant success rate in adults with ectodermal dysplasia [[28], [29], [30], [31], [32]]. There is still debate over when dental implants should be placed in developing toddlers. This study aims to evaluate the possibility of carrying out an implant therapy and the success rate of this therapy.

## 2 Materials and methods

### 2.1 Eligibility criteria

All documents were assessed for eligibility based on the following Population (including animal species), Exposure, Comparator, and Outcomes (PECO):

P) Participants consisted of patients.
E) Exposure consisted of patients with epidermolysis bullosa with an already established genetic diagnosis and treated with implant therapy.
C) The comparison was healthy patients with no history of EB or any significant medical diseases treated with implant therapy.
O) The outcome was to evaluate the survival of dental implants in patients with EB.

The secondary outcome consisted of evaluating the improvement of the quality of life through questionnaires in patients with EB after therapy with dental implants.

Only papers providing data at the end of the intervention were included. Exclusion criteria were: 1) Studies on EB patients who have not performed implant therapy; 2) Studies with groups of patients suffering from other systemic diseases so as not to alter the survival results of dental implants; 3) cross-over study design; 4) studies written in a language different from English; 5) full-text unavailability; 6) studies involving animal; 7) review article; 8) case report.

### 2.2 Search strategy

The primary scientific databases (PUBMED, WEB of SCIENCE, LILACS) were used to conduct the study (Table 1). The time taken into account for the electronic search was from January 3, 2000, to January 9, 2022. The term “Epidermolysis bullosa” has been combined with “dental implant.”

**Table 1:** Search strategy.

***PubMed*** (“epidermolysis bullosa” OR “EB”) AND (“dental implant” OR “oral implant”) AND (“oral health” OR “dentistry”)
***Lilacs*** TITLE-ABS-KEY (“epidermolysis bullosa” OR “EB”) AND (“dental implant” OR “oral implant”) AND (“oral health” OR “dentistry”)
***Web of Science*** (“epidermolysis bullosa” OR “EB”) AND (“dental implant” OR “oral implant”) AND (“oral health” OR “dentistry”)

The web search was assisted using MESH (Medical Subjects Headings).

This review was conducted using the PRISMA criteria. The study was registered, and the details were entered into PROSPERO with the identification number CRD42022356677.

### 2.3 Data extraction

Two reviewers (R.F. and G.M.) separately extracted data from the included studies using an individualised data extraction on a Microsoft Excel sheet. When there was a dispute, a third reviewer helped to achieve a consensus (L.F.).

The following data were extracted:1) First author; 2) Year of publication; 3) Nationality; 4) Age of study participants; 5) Number of dental Implants; 6) Implant survival rate; 7) Evaluation of implant survival over time.

### 2.4 Quality assessment

Using the Cochrane risk-of-bias tool for randomized trials, Version 2, two reviewers evaluated the publications' bias risk (RoB 2). Any discrepancy was handled with a third reviewer until an agreement was obtained.

## 3 Results

### 3.1 Study characteristics

**Fig. 1:**
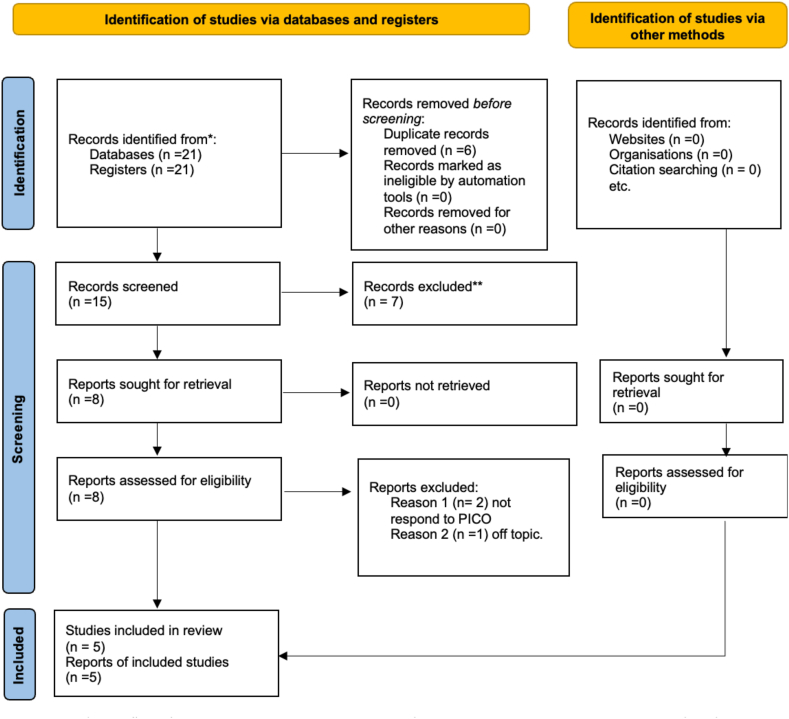
Prisma flowchart.

Twenty-one studies were found after the investigation. According to the PRISMA 2020 flowchart in Fig. 1, only five were chosen to create the current systematic study; 16 articles were skipped over. 10 papers were disregarded because they have been reviewed; 4 were ignored because they contained case studies; and two were omitted because they were not written in English. According to the PICO model, the remaining papers were chosen for title and abstract screening. Finally, five articles in the publication were found through search engines. In addition, a manual search of search engines such as EMBASE and Cochrane was performed. However, no matching clinical trials were found. A search of the bibliography was performed at the same time.

### 3.2 Main findings

Penarrhoca-Oltra's study evaluated the survival of the implant, the condition of the peri-implant tissue, and the improvement of the quality of life in a group of patients with dystrophic epidermolysis bullosa, all rehabilitated with a complete implant. Thirteen patients with dystrophic epidermolysis bullosa were enrolled and received implant treatment between 2005 and 2016. All these patients were recalled, and implant survival, tissue status, and patient satisfaction were evaluated. A total of eighty implants were inserted, of which forty-two were in the maxilla and thirty-eight in the mandible. The age range of the patients varies between 20 and 52 years, and all are diagnosed with dystrophic epidermolysis bullosa. After the patients' recall, an implant survival rate of 97.5% was evaluated after 7.5 years from the insertion of the prosthesis. More than 50% of the implants showed mucositis. The probing depth at the implant level of 1–3 mm was found at 96.2%, and bleeding on probing was appreciated at 67.5%. In 85% of the subjects, there was an abundant presence of bacterial plaque [33].

The study of Penarrocha M. aims to compare the well-being of rehabilitation of an edentulous arch with fully fixed implant-supported or removable implant-supported prostheses in a group of patients affected by dystrophic epidermolysis bullosa. Six patients were enrolled and treated with implant therapy. All patients had full edentulous mandibular, or maxillary. Three patients were treated with fixed prostheses, and three with removable prostheses stabilized with implants. Six months after the insertion of the prosthesis, the patients were given a questionnaire to assess their chewing and psychological well-being. The total number of implants inserted was thirty-eight, of which twenty-one were in the maxilla and seventeen in the mandible. The implant survival rate was 97.9%. The mean follow-up period was 5.5 years. Both fixed and mobile prosthetic rehabilitation has led to an improvement in the life of patients. The level of satisfaction was higher in patients rehabilitated with fixed prostheses supported by implants [34].

The Pennarhoca-Oltra study evaluated a bone graft's implant survival rate and simultaneous success rate to support implant therapy. The retrospective study evaluated four patients treated with implant therapy and simultaneous particulate bone grafting between 2005 and 2009. All patients with dystrophic epidermolysis had repercussions at the oral level and were rehabilitated with fixed prostheses. Eighteen implants had thread fenestration during the surgical phase and were treated with particle bone grafts; fourteen received autologous bone grafts, and four tricalcium beta-phosphate implants. In sixteen implants, the graft has been covered with a resorbable collagen membrane; in two implants, the graft has been covered with a titanium membrane. All implants survived in the mean follow-up period of 12 months [35].

Pennarocha-Oltra's study evaluated survival and increased quality of life of patients rehabilitated with fixed prostheses supported by four implants. A retrospective study assessed the quality of life in patients treated with four implant-fixed prostheses. Thirty-two implants were inserted to support eight fixed full-arch prostheses, of which 20 implants were inserted in the maxilla and 12 in the mandible. Implant survival with a mean follow-up of 22.9 months after prosthesis insertion was 100%. Patient satisfaction after implant therapy was extremely high [36].

Penarrocha-Diago's study is a retrospective study that evaluated four patients with dystrophic epidermolysis bullosa undergoing implant therapy. Patients in the study showed significant oral soft tissue involvement. Fifteen implants were inserted, respectively 7 in the maxilla and 8 in the mandible. All implants are successfully integrated and restored. The mean follow-up of the implants was 2.5 years [37] (Table 2).

**Table 2:** Main characteristics of the studies included in the present systematic review.

Articles	Samples	Results	Clinical significance
Peñarrocha-Oltra, 2020	13 patients with dystrophic epidermolysis bullosa were enrolled and underwent implant treatment between 2005 and 2016	After the recall of the patients, an implant survival rate of 97.5% was evaluated 7.5 years after the insertion of the prosthesis. Of most of the implants, about 50% showed mucositis at the time of follow-up.	The quality of life improves. Implant survival is similar to the rest of the population.
Peñarrocha, 2007	Six patients were enrolled and treated with implant therapy. The patients all had complete edentulous or mandibular, or maxillary	Three patients were treated with fixed screw-retained prostheses, and three were treated with implant-supported removable prostheses. The implant survival rate was 97.9%. The mean follow-up period was 5.5 years	The quality of life improves. Implant survival is similar to the rest of the population.
Peñarrocha-Oltra, 2012	Four patients treated with implant therapy and simultaneous particulate bone grafting	All implants survived in the mean follow-up period of 12 months	The quality of life improves. Implant survival is similar to the rest of the population.
Peñarrocha-Oltra, 2011	32 implants were inserted to support eight fixed full-arch prostheses	Implant survival with a mean follow-up of 22.9 months after prosthesis insertion was 100%.	The quality of life improves. Implant survival is similar to the rest of the population.
Peñarrocha-Diago, 2000	Four patients with EB. 15 implants were inserted	All implants are successfully integrated and restored. The mean follow-up of the implants was 2.5 years	The quality of life improves. Implant survival is similar to the rest of the population.

### 3.3 Quality assessment and risk of bias

**Fig. 2:**
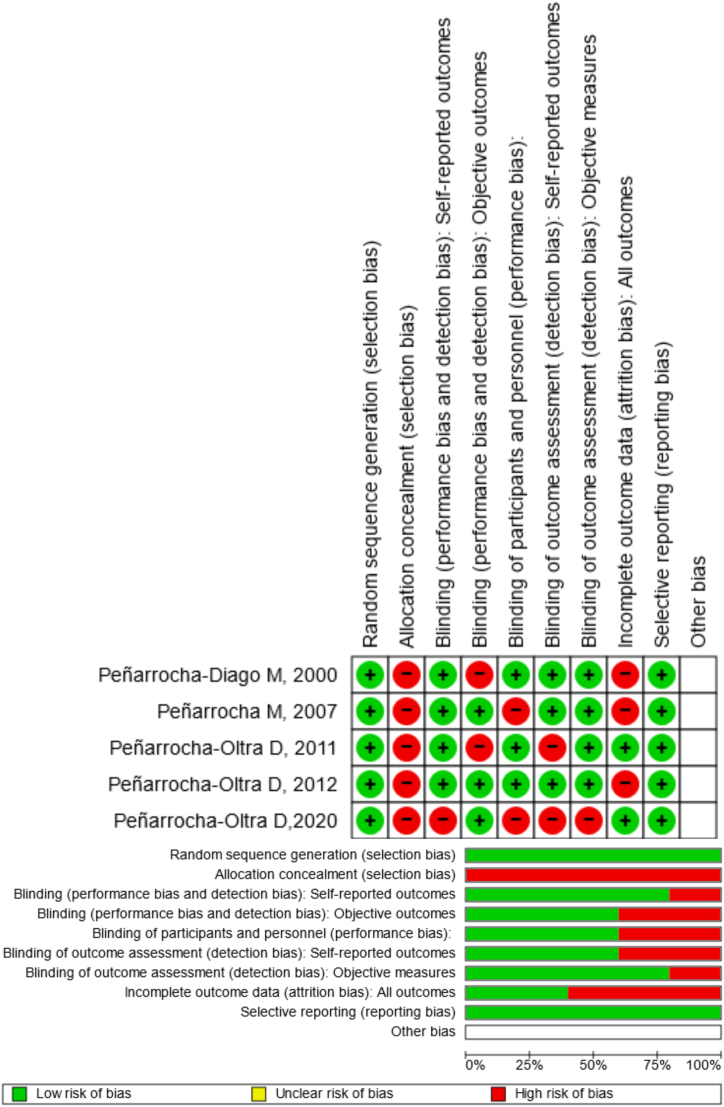
Risk of bias domains of the included studies.

The risk of bias was calculated using the RoB 2 and displayed in Fig. 2. Concerning randomization, a low risk of bias was guaranteed by all trials. Regarding allocation concealment, all articles had a substantial risk of bias. The results of the self-reported study revealed that 80% had a minimal risk of bias. The objective results, blinding of participants and staff, and blinding of outcome evaluation showed little chance of bias in 80% of the trials. A 40% low probability of bias is indicated by the papers' incomplete outcome data (objective measures). Regarding selective reporting, there was little chance of bias in every study.

## 4 Discussion

Dental implants have revolutionized the field of dentistry by offering a highly effective and aesthetically pleasing solution for the replacement of missing teeth. Osseointegration, the direct structural and functional connection between the dental implant and surrounding bone, is crucial in the success and long-term stability of dental implant procedures. This comprehensive review explores the concept of osseointegration in dental implants, including its biological mechanisms, factors influencing osseointegration, clinical applications, and future directions in the field. Dental implants have become the gold standard for tooth replacement, with a success rate of approximately 95% over ten years. Osseointegration, a term first introduced by Professor P.I. Brånemark in the 1960s, is the cornerstone of dental implantology. It describes the direct bonding between the implant surface and the surrounding bone, allowing for functional and long-lasting prosthetic restorations. This review will delve into the intricacies of osseointegration, its underlying mechanisms, influencing factors, clinical applications, and emerging trends in dental implantology.

Osseointegration is a complex biological process involving the implant's surface, bone tissue, and the host response. The sequence of events can be summarized as follows:

• **Implant Surface:** Dental implants are typically made of biocompatible materials like titanium. Their surface properties, including roughness, chemistry, and topography, play a significant role in osseointegration. A rough surface promotes better cell adhesion and proliferation, forming a robust bone-implant interface.
• **Blood Clot Formation:** A blood clot forms around the implant after implant placement. Platelets in the clot release growth factors, such as platelet-derived growth factor (PDGF) and transforming growth factor-beta (TGF-β), which initiate the healing cascade.
• **Inflammatory Phase:** Inflammatory cells infiltrate the site, mainly neutrophils and macrophages. This phase is essential for removing debris and preparing the tissue for osteogenic processes.
• **Osteogenic Phase:** Osteoblasts, bone-forming cells, become active and secrete extracellular matrix proteins, creating a mineralized bone matrix. This phase is characterized by the deposition of hydroxyapatite on the implant surface.
• **Maturation:** Over time, the newly formed bone matures and remodels. The implant fully integrates with the surrounding bone, leading to a stable and functional prosthetic restoration.

Clinical manifestations can externalize the oral mucosa and the teeth' structure, shape, and number. It becomes essential to rehabilitate this type of patient through implants as they are much more subject to agenesis. Many have questioned the actual safety and efficacy of implant therapy [23,38]. The interest has shifted towards evaluating implant survival in patients with epidermolysis bullosa to restore their chewing and improve their quality of life [[39], [40], [41], [42], [43]]. Patients with EB who experience oral complications may struggle to chew and swallow food, consume enough nutrients, and may feel less confident due to their altered appearance and speech issues.

Consequently, a multidisciplinary strategy is necessary for medical and dental treatment. Dental care requires carefully executed, minimally traumatic procedures. Patients' general health influences their willingness to comply in the dental office and at home, as well as their oral hygiene and nutrition. To maintain practical masticatory function, clinicians must administer conservative and restorative treatments because doing so lowers the chance of developing oral and oesophagal lesions [44].

Given the trouble in opening the mouth and the brittleness of the epithelial tissues, extra caution is required. Specific physiotherapy movements are advised for patients with restricted tongue mobility or difficulty opening their mouths. Researchers advise using lubricants (such as petroleum jelly, glycerin, and hydroxyethyl cellulose, methyl cellulose) on the patient's lips and oral mucosa as well as on gloves, radiographic film, and tools to lower the risk of blister development. The risk of developing caries is raised by several EB-related factors [45]. First, swallowing is challenging; kids usually consume small amounts of pureed food throughout the day. Slow clearance increases the time that food and beverages are in touch with the teeth. Doctors frequently add caloric, high-carbohydrate drinks to their patients' diets to increase calorie consumption. Therefore, researchers highly advise patients with these conditions to use pit-and-fissure sealants. Patients with EB experience trouble brushing their teeth because blistering of the hands and fingers diminishes their ability to hold a toothbrush. Brushing can be challenging due to microstomia and oral scarring, and oral care practices are restricted due to concern over developing new lesions. Family members and caretakers should be involved in the supervision of effective, trauma-free brushing and adherence to dietary advice. Mouthrinses ought to be flavourless and devoid of alcohol.27 The most effective dental antiseptic is chlorhexidine, best used as a mouthwash at concentrations of 0.12–0.2%. Microstomia and ankyloglossia may make rinsing challenging for patients with significant oral pain; in this case, a 0.2% chlorhexidine spray can be used instead. While acidic fluoride preparations hurt when oral ulceration develops, neutral pH sodium fluoride mouth rinses are helpful [46]. If fluoride is absent in the drinking water, Wright and colleagues advised patients to obtain systemic fluoride supplements and topical fluoride varnish applications every three to four months. It is not required to use compounds made of resin. Restorations must be free of sharp edges or potentially harmful surfaces, and restorative materials must be polished and finished correctly. Typical healing times for lesions brought on by trauma during dental restorative treatments are one to two weeks, with no special care required. It is proven that preformed steel crowns are essential for pediatric patients.

Endodontic therapy is no longer considered contraindicated, although tooth extraction was once the preferred option for patients with caries affecting the pulp. Clinicians may encounter challenges when introducing tools due to microstomia and restrictions on mouth opening. Krämer recently published a report on nine patients with RDEB who underwent a succession of successful endodontic procedures. Most EB patients tolerate orthodontic therapy well, but it's crucial to wax brackets to lessen soft-tissue damage. To treat malpositioned teeth in DEB patients, some writers have suggested using serial tooth extraction [47].

All EB patients, including those with DEB who have periodontal disease, require periodontal therapy. However, medical professionals need to treat soft tissues with the utmost care. Although ulcers may develop due to intraoral manipulation, they typically go away independently in one to two weeks without medical attention. We could not locate any agreement in the research regarding the use of sutures. Some medical professionals avoid using them to prevent sores from forming, while others claim that suturing has never caused any problems. Most EB patients tolerate orthodontic therapy well, but it's crucial to wax brackets to lessen soft-tissue damage. To treat malpositioned teeth in RDEB patients, some writers have suggested using serial tooth extraction.

All EB patients, including those with RDEB who have periodontal disease, require periodontal therapy. However, medical professionals need to treat soft tissues with the utmost care. Although ulcers may develop due to intraoral manipulation, they typically go away independently in one to two weeks without medical attention. We could not locate any agreement in the research regarding the use of sutures. Some medical professionals avoid using them to prevent sores from forming, while others claim that suturing has never caused any problems. Patients suffering from epidermolysis bullosa have manifestations at the level of the oral mucosa, with blistering, which in severe cases can compromise chewing and feeding due to the formation of scars at the oral level. In addition, dental elements are also involved; therefore, implant therapy to restore lost chewing and improve the quality of life in this category of patients has become mandatory [48,49]. This review examined the articles in the literature to verify whether implant therapy for simple edentulous and rehabilitating complex edentulous arches is a reliable therapy with a guarantee of survival [48,50]. The use of implant therapy is now well established in the world population, and therefore, its benefits could be helpful in this category of patients. This study aimed to bring together all clinical cases of implant therapy in patients with epidermolysis bullosa. Even after implant therapy, however, the patients showed ulcerations and problems in the mucous membrane of the oral cavity due to food, brushing, and all causes of friction at the level of the oral cavity [[50], [51], [52], [53]]. All the studies considered have shown, even if the presence of ulcerations, an increase in the quality of life of patients with EB. In fact, before implant rehabilitation, most patients showed difficulty chewing and were forced to ingest semiliquid foods to avoid friction and ulceration at the oesophageal level. All patients surveyed reported improved chewing after implant rehabilitation, claiming to chew solid foods. However, the implant failure rate in the studies is about 1.3% on average [[54], [55], [56]]. The implant failures occurred early or during the second surgery (primary failures). The scientific literature states that the main implant failures occur early, regardless of the follow-up period. The follow-up of these reviewed studies is short and does not exceed four years. Therefore, the failure rate is underestimated, as late failures may occur, but according to the literature, they are the bottom of all kinds of losses [56].

Multisystemic epidermolysis bullosa has a significant mortality rate in some variants and various co-morbidities [57]. A multidisciplinary team that works closely together must address patients with EB holistically. DEB is a severe variation of EB that frequently manifests orally. Early bucco-dental follow-up makes preventing and treating oral decay easier, which is frequently unavoidable. Although osseointegration and bone repair are unaffected by this mucocutaneous illness, a bone graft can be done before implant placement. However, as jugal synechiae limits prosthetic space generally, this must be considered. It is advised to use Therabite therapy or oral opening exercises. A vestibuloplasty that permits a partial debridement of the synechiae may be considered to improve this region.

Implant-based prosthetic rehabilitation appears to be a promising approach. Research demonstrates that DEB does not preclude implants or prosthetic rehabilitation. Only two implants out of 161 implanted in research involving 28 patients failed. A further investigation of 38 dental implants revealed a 97.9% success rate. Some dental recommendations for the treatment of patients with EB have been drawn up: the aspirator should be in contact only with bones or teeth and never with soft tissues to avoid ulceration; always lubricate the lips or mucous membranes, for example, with petroleum jelly; if possible, the incisions with the blade should be minimized to avoid subsequent blisters [34,35,[58], [59], [60]]. All the studies agree that the implant success rate is like the rest of the Population; however, as already specified, it is a concise survival rate due to limited follow-up [61]. This study's results agree with case reports in the literature that have analyzed and evaluated performance and failure rates. All agree with implant therapy's excellent efficacy in patients with epidermolysis bullosa [60]. Panadero's case series evaluated implant survival in patients with EB. The study was not considered because of the case series [62].

Moreover, it agrees with our results, predicting implant survival close to 100%. It also recommends using the intraoral scanner for impression taking as it is less traumatic and avoids the formation of ulcers due to the pathology itself [[63], [64], [65]]. Therefore, it was deemed essential to do a systematic review of the literature on implant survival, as intake and chewing are essential in patients with EB since food trauma could notch and create severe ulceration. In addition, the studies have given some clinical guidance on how patients can be clinically managed, such as ‘fingerprinting. In addition, reestablishing mastication in this group of patients is very important because the trauma given by improperly chewed food could aggravate the pathology.

### 4.1 Limitation of study

The limitation of this study is that studies by the authors themselves were considered. Furthermore, these are not randomized studies, so the implant success rate of the rest of the Population was taken from the studies in the literature. The Peñarrocha-Oltra et al., 2020 study is the only retrospective study in which it was stated that patients taken from the previous studies were considered. Therefore, The observation period is more extended, so long-term implant survival was evaluated, although the patients are the same with the same comorbidities. In the literature, even through a manual search, there are only case reports; therefore, they were not considered. The small number of scientific articles about this topic currently represents the limitation of the study. In this review, we considered only clinical trials or randomized clinical trials; therefore, the Population is small, with the risk of an overlapping population. However, only the study by Peñarro-cha-Oltra D (2020) did a retrospective study in which they stated that they considered part of the patients from the studies considered. However, we also analyzed case reports and case series, and they agree with our results. So, although with the limitation of data, we can say that implant therapy is effective and free of systemic contraindications. A reference list analysis with the Fi-index Tool has been performed: This manuscript has been checked for the first author with the Fi-index Tool with a score of 2.34 on October 20, 2022, according to Scopus®.

## 5 Conclusions

Implant therapy improves the quality of life of patients affected by this pathology, and implant therapy is a reliable surgery with a similar success rate to the rest of the Population. Further studies will be needed to confirm this hypothesis and with a longer follow-up.

## Availability of data and materials

The data will be available on reasonable request from the corresponding author.

## Funding

This research received no external funding

## CRediT authorship contribution statement

**Giuseppe Minervini:** Formal analysis, Data curation, Conceptualization. **Rocco Franco:** Funding acquisition, Formal analysis, Data curation, Conceptualization. **Maria Maddalena Marrapodi:** Visualization, Validation, Supervision, Conceptualization. **Antonino Lo Giudice:** Writing – review & editing, Writing – original draft, Visualization, Validation, Supervision, Software, Conceptualization. **Marco Cicciù:** Writing – review & editing, Writing – original draft, Visualization, Validation. **Vincenzo Ronsivalle:** Writing – review & editing, Supervision, Software, Resources, Project administration, Methodology, Investigation, Funding acquisition.

## Declaration of competing interest

The authors declare that they have no known competing financial interests or personal relationships that could have appeared to influence the work reported in this paper.
